# Supplementary material for: Non-Invasive Quantification of Faecal and Urine Reproductive Hormone Metabolites in the Naked Mole-Rat (Heterocephalus glaber)
Source: Animals (Basel). 2023 Sep 27;13(19):3039. doi: 10.3390/ani13193039 (PMC10571929; doi:10.3390/ani13193039)
Supplement: Supplementary file 1 [file animals-13-03039-s001.zip › animals-2614113-supplementary.pdf]

Supplementary Materials

Table S1. Details on EIA antibodies, label, standard, and references for each EIA used in the study.

Name	Antibody raised against	Label	Standard	EIA described in
**5α-Progesterone** *(P9-IZW)*	5α-pregnane-3β-ol-20-one-3-HS-BSA	5α-pregnane-3α-ol-20-one -3-HS-HRP	5α-pregnane-3α-ol-20-one	[32]
**Progesterone** *(P4-Ak6, 5β-20-one)*	5α-pregnane-3α-ol-20-one-3HS:BSA	5α-pregnane-3α-ol-20-one-3HS:DADOO-biotin	Progesterone	[33]
**Testosterone** *(T-3-CMO)*	Testosterone-3-CMO:BSA	5α-androstane-3β,17β-diol-3-HS:DADOO-biotin	Testosterone (17β-Hydroxy-3-oxo-4-androstene)	[34]
**Epiandrosterone**	5α-androstane-3α-ol-17-one-HS:BSA	5α-androstane-3,17-dione-thioether: DADOO-biotin	Epiandrosterone (5α-androstan-3β-ol-17-one)	[34]

DADOO-biotin = N-biotinyl-1,8-diamino-3,6-dioxaoctane; HS = hemisuccinate; HRP = horseradish peroxidase; CMO = carboxymethyloxime; BSA = bovine serum albumin

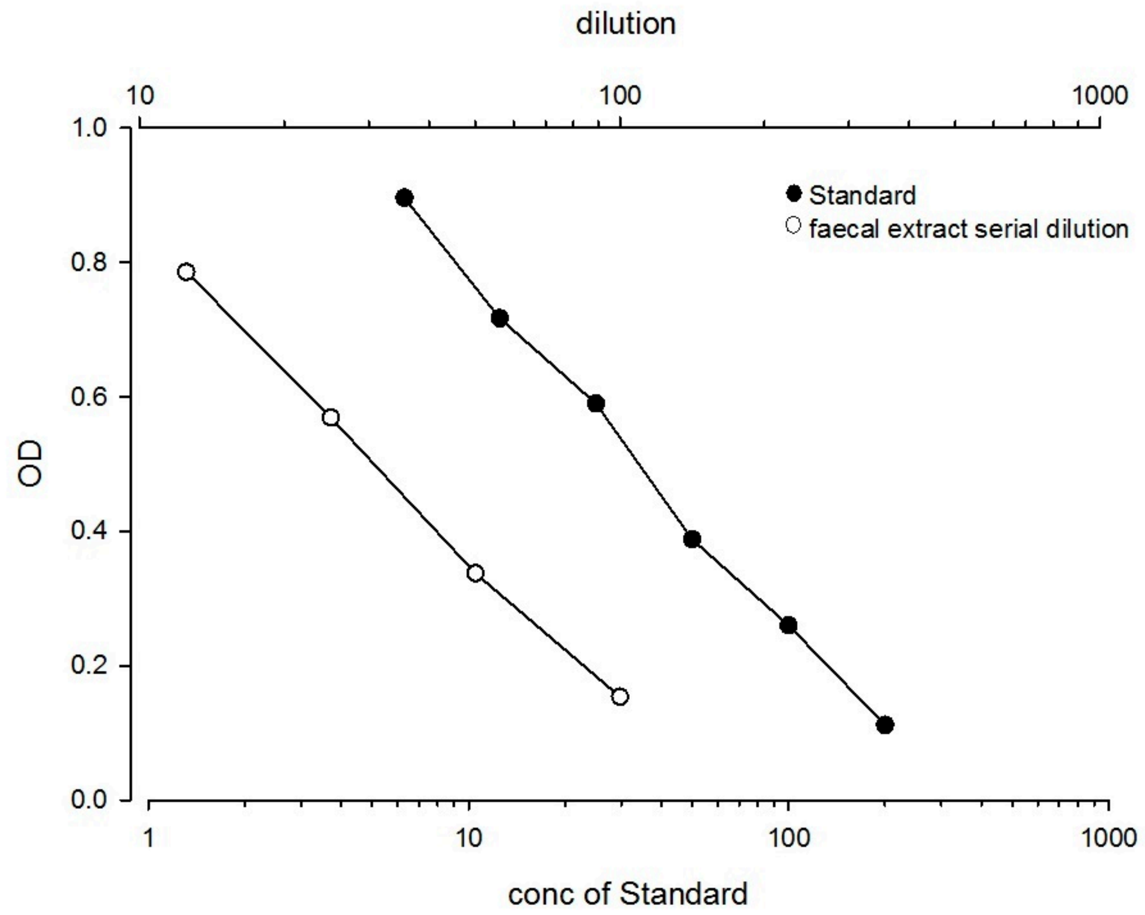

Figure S1. Serial dilutions of faecal extracts for the 5α-Progesterone EIA.

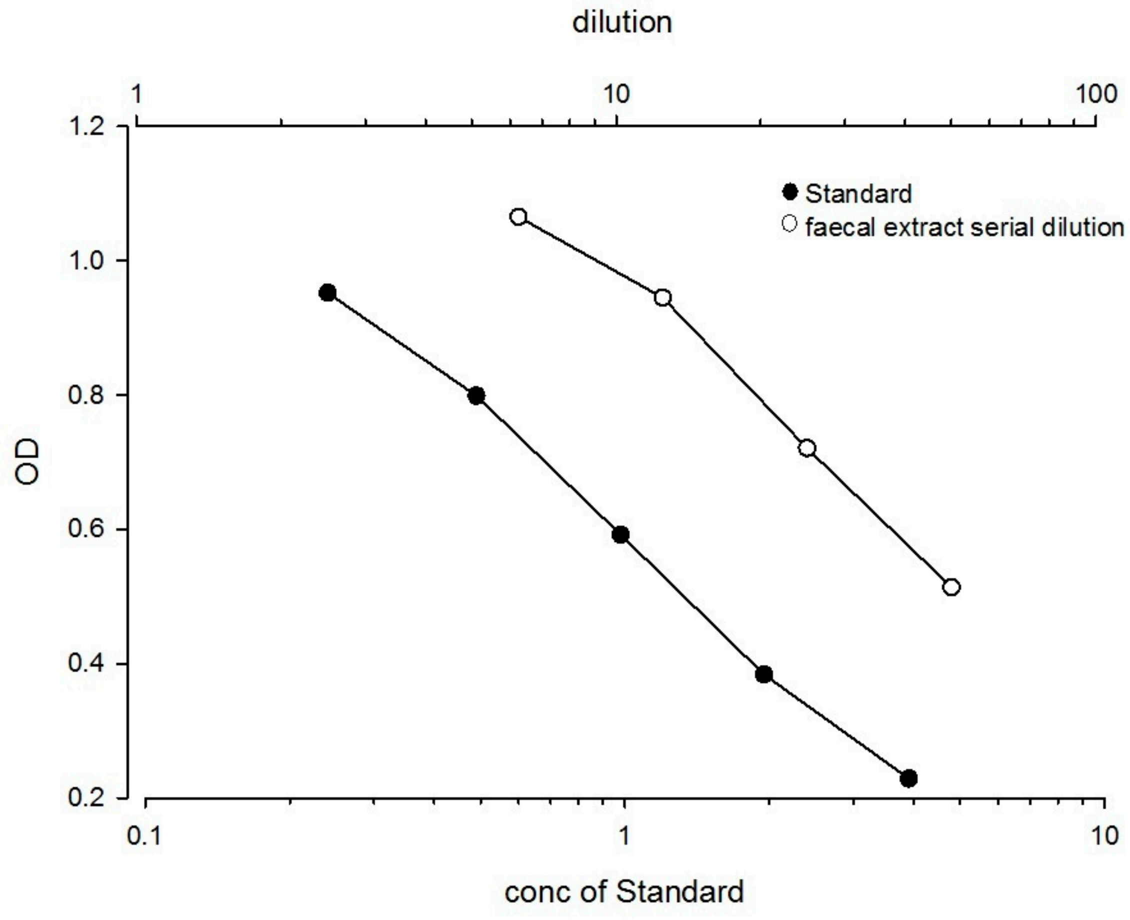

Figure S2. Serial dilutions of faecal extracts for the Epiandrosterone EIA.
